# Editorial: Purinergic signalling in the central nervous system and its pharmacological importance in neurological and psychiatric illnesses

**DOI:** 10.3389/fphar.2023.1355286

**Published:** 2024-01-12

**Authors:** Yong Tang, Patrizia Rubini, Beata Sperlagh, Henning Ulrich

**Affiliations:** ^1^ International Joint Research Centre on Purinergic Signalling, Chengdu, China; ^2^ Acupuncture and Chronobiology Key Laboratory of Sichuan Province, School of Health and Rehabilitation, Chengdu University of Traditional Chinese Medicine, Chengdu, China; ^3^ Laboratory of Molecular Pharmacology, HUN-REN Institute of Experimental Medicine, Budapest, Hungary; ^4^ Department of Biochemistry, Institute of Chemistry, University of Sao Paulo, Sao Paulo, Brazil

**Keywords:** purinergic signalling, central nervous system, neurological and psychiatric illnesses, ATP, P1, P2, adenosine

This Research Topic aims to honour the 80th birthday of Professor Peter Illes, who is a member of the European Academy of Sciences, the founder/first president of the German Purine Club, and honorary president of the Chinese Purine Club. His connections with China explain that a number of Chinese scientists contributed with articles to this Research Topic. Peter Illes established a worldwide co-operation network on purinergic signalling and is an internationally recognized leader in the field of his discipline.

Adenosine Triphosphate (ATP) is an intracellular energy-storing molecule, but may also reach the extracellular space, where it participates in cell-to-cell signalling. For this purpose, ATP utilises a range of purinergic receptors activated either by ATP itself (P2X receptors; seven subtypes) or by ATP/ADP and UTP/UDP (P2Y receptors; eight subtypes) and finally via its enzymatic degradation product, adenosine (P1/A receptors; four subtypes). Purine nucleotides and nucleosides together with the whole plethora of receptors and degrading enzymes constitute the purinome. This fascinating and extensive network exists both in animals and humans and is essential in regulating important physiological functions. Disturbances in the network can lead to a variety of illnesses clinically associated with both neurological or psychiatric traits. In recent years, hope has arisen that pharmacology and medicinal chemistry together with various newly developed methods, will enable researchers to discover and design efficient drugs for treating these neurodegenerative and affective illnesses, based on disturbances of the purinergic system. Although this Research Topic clearly concentrates on brain diseases due to misbalance and inefficiency of the purinome, it has to be pointed out that a wide range of diseases arise because of the same reason in all parts of the human body, which is however no subject of the present discussions.

In this Research Topic, seven original articles, one mini-review, and two overviews were included. Of three review papers, one focuses on the potential application of P2Y12 receptor (R)-ligands in the diagnosis and treatment of epilepsy (Chen et al.), another two summarize the roles and neurobiological mechanisms of adenosine and its receptors in sleep-wake regulation, torpor and hibernation (Ma et al.), as well as the role of purinergic signalling in the modulation of blood-brain barrier (BBB) permeability (Wang et al.), respectively.

In the following we will enumerate in a one sentence synopsis the content of the individual articles included in our Research Topic. 1) The adenosine receptor-subtype A2A was identified to exert distinct control on the morphology of retinal ganglion cells (RGC), in order to cell-type specifically fine-tune the RGC dendritic morphological complexity during normal development and neonatal inflammation (Hu et al.). 2) P2X3 receptors in the dorsal root ganglion and spinal cord have been shown to mediate the analgesic effect of Polyphyllin VI (Luo et al.), known to be responsible for cell-cycle arrest and acupuncture efficiency. 3) Diclofenac was demonstrated, by the application of two-electrode voltage clamp electrophysiology, to be a strong antagonist of the human (h)P2X3 and hP2X2/3Rs, but a weaker blocker of hP2X1, hP2X4, and hP2X7Rs (Grohs et al.). 4) Morphotyping and single cell shape descriptor analysis demonstrated that in cultured microglia, treatment with the P2X7R agonist dibenzoyl-ATP (Bz-ATP), and the inflammatory activator lipopolysaccharide (LPS) in combination with Bz-ATP, increased round/ameboid microglia and decreased polarized ramified morphology of this cell type (von Mücke-Heim et al.). 5) Cytokines appear to play important roles in the antidepressant-like effect of zinc in male mice (Iring-Varga et al.). 6) A small nucleotide polymorphism (SNP) analysis performed in epileptic patients indicated that the TT genotype and T allele of rs4431401 in CD73 (ecto 5′-nucleotidase, generating adenosine from AMP) were genetic risk factors for epilepsy in male patients, whereas rs2267076, rs2298383, rs4822492, and rs4822489 polymorphisms of the A2AR were mainly associated with female subjects (Shi et al.). 7) Finally, eicosapentaenoic acid (EPA) was described to be an inhibitor of the vesicular nucleotide transporter (VNT), which actively transports nucleotides into secretory vesicles responsible for the storage of ATP. VNT certainly appears to plays an essential role in purinergic transmission, although it is still premature to conclude that EPA is a specific inhibitor of this enzyme (Moriyama et al.).

In this Research Topic, hP2X1, hP2X3, hP2X2/3, hP2X4, and hP2X7Rs, as well as the human SNPs of CD73 and A2ARs were investigated in diverse studies. It was an interesting finding that in male and female mice, different SNPs cause predisposition to epilepsy. However, only future studies will decide which of the reported data have major significance for clinical practice.

